# Pharmacoresistant Severe Mental Health Disorders in Children and Adolescents: Functional Abnormalities of Cytochrome P450 2D6

**DOI:** 10.3389/fpsyt.2018.00002

**Published:** 2018-01-24

**Authors:** Susanne Thümmler, Emmanuelle Dor, Renaud David, Graziella Leali, Michele Battista, Alexia David, Florence Askenazy, Céline Verstuyft

**Affiliations:** ^1^University Department of Child and Adolescent Psychiatry, Children’s Hospitals of Nice CHU-Lenval, Nice, France; ^2^CoBTek, Université Côte d’Azur, Nice, France; ^3^Department of Child Psychiatry, Nice Children’s Hospitals CHU-Lenval, Nice, France; ^4^Department of Child and Adolescent Psychiatry, Hospital of Fréjus, Fréjus, France; ^5^Service de génétique moléculaire, pharmacogénétique et hormonologie, Centre de Ressource Biologie Paris-Sud, Hôpital Bicêtre, Groupe Hospitalier Paris Sud, AP-HP, Le Kremlin Bicêtre, Nice, France; ^6^Université Paris-Sud, UMR 1184, Faculté de médecine, Paris, France

**Keywords:** pharmacogenetics, personalized medicine, antipsychotics, antidepressants, child and adolescent psychiatry, CYP2D6

## Abstract

**Background:**

Severe mental health disorders in children and adolescents represent a major public health problem. Despite adequate drug treatment, some patients develop pharmacoresistant disease. As a consequence, physicians are confronted with prescribing challenges, prolonged hospitalization and increased risk of adverse events, thus aggravating short-, medium-, and long-term prognosis. The majority of psychotropic treatments, particularly antipsychotics and antidepressants, are metabolized at hepatic level by cytochrome P450 (CYP), particularly by CYP3A4 and CYP2D6. Several *CYP2D6* genetic polymorphisms are described to be associated with ultrarapid (UM) or poor drug metabolism (PM), inducing clinical resistance and/or adverse events, and might therefore be related to pharmacoresistant severe mental health disease.

**Case presentation:**

A total of nine pharmacoresistant patients (four females, five males) aged 11–16 (mean 14.1) years have been genotyped for *CYP2D6* between January, 2015 and April, 2016. Patients were diagnosed with schizophrenia (*n* = 5), autism spectrum disorders (*n* = 2), intellectual disability with challenging behavior (*n* = 2), oppositional defiant disorder (*n* = 1), and post-traumatic stress and borderline personality disorders (*n* = 1). They had a treatment history with on average 6.1 (3–9) psychotropic, 5 (3–7) antipsychotic, and 3.4 (2–5) CYP2D6-metabolized antipsychotic and antidepressant molecules. Five patients (56%) presented functional anomalies of the *CYP2D6* gene: three patients were UM metabolizers with gene duplication and two patients were PM with *4/*41 and *3/*4 polymorphisms.

**Conclusion:**

Functional anomalies of *CYP2D6* concerned more than half of our pediatric inpatient sample with pharmacoresistant disease. However, our case reports are limited by the low sample size. Nevertheless, knowledge of individual metabolism and in particular *CYP2D6* genotyping should be considered for clinical workup and therapy adjustment in resistant patients in child and adolescent psychiatry and might permit better treatment outcome, increased treatment adherence and diminished adverse events.

## Background

Pharmacoresistant severe mental illness is an important public health burden, especially in children and adolescents with chronic disabling disease. Treatment decisions in those children and adolescents are generally challenging and many of these patients are hospitalized in inpatient children and adolescent psychiatry (CAP) departments at one point of their illness. Clinicians are therefore in the need of complementary explorations aiming to optimize treatment in order to increase the probability of clinical efficacy and to minimize eventual adverse events, improving treatment adherence and long-term outcome.

Cytochromes P450 (CYP450) are proteins implicated in metabolism and expressed in different organs such as liver, gut and brain (1, 2). There are about 18 families of CYP450 with CYP1, CYP2, and CYP3 most involved in drug metabolism, as well as different subfamilies (e.g., CYP1A, CYP3A, and CYP2D) and isoenzymes (e.g., CYP1A2, CYP3A4, and CYP2D6).

The CYP2D6 enzyme is of special interest for child and adolescent psychiatrists. At hepatic level, CYP450 enzymes are responsible for about 80% of all phase I metabolism reactions. Even though CYP2D6 constitutes only 2% of all CYP450 enzymes expressed at the hepatic level, it is involved in the metabolism of about 20% of drugs (1), and especially of psychotropic drugs frequently used in CAP (see also Table 1).

**Table 1:** Examples of CYP2D6 metabolism or interaction of antipsychotic and antidepressant medications used in Child and Adolescent Psychiatry.

Substrates of CYP2D6 Antipsychotics	Antidepressants
Risperidone	Sertraline
Aripiprazole	Fluoxetine
Quetiapine	Citalopram
Pimozide	Escitalopram
Haloperidol	Venlafaxine
Levomepromazine	Paroxetine
Zuclopenthixol	Amitryptiline
**Inhibitors of CYP2D6**	
Risperidone	Fluoxetine
Haloperidol	Paroxetine
Levomepromazine	

*References: http://www.hug-ge.ch/sites/interhug/files/structures/pharmacologie_et_toxicologie_cliniques/a5_cytochromes_hd.pdf; Spina and de Leon (3)*.

The CYP2D6 and CYP2C19 are highly polymorphic with over 100 allelic variants identified for CYP2D6^1^ and over 30 allelic variants for CYP2C19^2^ (4, 5).

Phenotypes of the CYP2D6 activity, resulting from the analyses of these polymorphisms, are classified into extensive (EM), poor (PM), and ultrarapid (UM) metabolizers accordingly to the enzymatic activities (6). The most common EM have normal CYP2D6 function. Decreased or no CYP2D6 function is observed in PM, and increased CYP2D6 function is related to UM. Individual CYP2D6 genotype and function also depend on ethnic origin. Polymorphisms leading to poor metabolization (PM) are present in about 3–10% of Caucasians (7–9). Gene duplication resulting in UM concerns about 1% of the general population in Northern European Caucasians and up to 10% in Southern Europeans (7) and might be as high 29% in African Ethiopians (10).

In addition, several studies underline that certain *CYP2D6* genotypes are associated with psychotropic treatment response as well as to the occurrence of adverse events in children and adolescents (11–14).

Despite the increasing use of pharmacogenetic (PGx) testing for treatment decisions in different fields of medicine, especially in oncology (15), CYP genotyping is only rarely used in French CAP practice.

In this case report, we present PGx results for CYP2D6 genotyping in an inpatient sample of pediatric individuals hospitalized in CAP and presenting severe mental illness with repeated psychotropic treatment failure. We hypothesized that functional anomalies of the *CYP2D6* gene, especially those leading to ultrarapid metabolism, might be present in some of these patients.

## Methods

A retrospective chart review was conducted in order to evaluate *CYP2D6* genotyping data from children and adolescents of the inpatient units of the University Child and Adolescent Psychiatry department, Nice, France, between January 2015 and April 2016. *CYP2D6* PGx has been introduced since January 2015 as a complementary analysis of inpatients presenting with pharmacoresistant mental health disease. The factors determining pharmacoresistance were multiple failed responses to psychotropic medications and polypharmacy. All patients/guardians gave written informed consent for CYP450 PGx testing and for the use of this data for research purposes and publication. The data collection has been declared to the French national commission on informatics and liberty (CNIL No. 1970115v0).

Patients were systematically genotyped for the major CYP2D6 genetic polymorphisms and classified into three CYP2D6 phenotype subgroups (PM, EM, and UM). Genomic DNA was extracted from circulating blood leukocytes by using Qiagen Blood Kits according to the manufacturer’s protocol (Qiagen, S.A., Courtaboeuf, France).

Patients were genotyped for the major alleles *CYP2D6*: loss of function alleles (CYP2D6 *3 rs35742686, *4 rs3892097, *6 rs5030655, *41 rs28371725); using TaqMan allelic discrimination (16) with the ABI Prism^®^ 7900HT Sequence Detection System (Applied Biosystem, Courtaboeuf, France). The complete deletion (CYP2D6*5) and gene duplication (CYP2D6 *2xN) were detected by quantitative PCR according to the methods of Ref. (17). We employed descriptive statistics using mean and SD.

## Case Presentation

A total of nine individuals, four females and five males, aged 11–16 years (14.1 ± 1.8) have been genotyped for CYP2D6. Patient characteristics are detailed in Table 2. Patients had a treatment history with the failure of 3– 9 psychotropic medications (6.1 ± 2.3) including 3–7 antipsychotics (5 ± 1.5), 2– 5 CYP2D6-metabolized molecules (3.4 ± 0.9), and 1–3 CYP2D6-inhibitor drugs (2.2 ± 0.83). Most patients were diagnosed with childhood-onset schizophrenia (COS, *n* = 5) with one also having been diagnosed with autism spectrum disorder (ASDs). Two patients presented with intellectual disability (ID) associated with severe behavioral disorder (BD) such as aggressive behavior. The other two patients have been diagnosed with ASD and oppositional defiant disorder (ODD), and posttraumatic stress disorder (PTSD) and borderline personality disorder (BPD).

**Table 2:** Patient characteristics and results of CYP2D6 genotyping.

No.	Age	Sex	Diagnosis	Treatments (substrates of CYP2D6 underlined)	Major adverse events	PGx testing of *CYP2D6*
1	16	M	COS	AP: RISP, HAL, LMZ, CLZ, OLZ, PCZ	Extrapyramidal syndrome, weight gain (RISP), hepatic cytolysis (CLZ)	UM (duplication)
				Other: BZD, VPA		

2	14	F	COS	AP: RISP, ARP, QTP, AMS, CLZ, CMZ, LOX	Numerous adverse events: extrapyramidal syndrome, akathisia, dystonia, galactorrhea, “binge eating,” weight gain, constipation	PM (*4, *41)

3	15	F	PTSD, BPD	AP: RISP, ARP, QTP, CMZ, PCZ	Weight gain, “binge eating,” CBZ overdosage [patient 1 (18)]	UM (duplication)
				AD: ESC, FLX		
				Other: BZD, CBZ		

4	15	M	COS	AP: RISP, ARP, QTP, AMS, CMZ	–	Normal, EM
				AD: SRT		

5	16	M	COS	AP: RISP, ARP, QTP, HAL, CMZ	–	UM (duplication)

6	13	F	ASD, ODD	AP: RISP, ARP, PMZ, AMS, CMZ, PCZ, CLZ	–	Normal, EM
				AD: SRT		
				Other: Li		

7	12	F	ASD, COS	AP: RISP, ARP, CMZ	Hepatitis (CMZ)	Normal, EM

8	11	M	ID, BD	AP: RISP, ARP, ZP	–	PM (*3, *4)

9	15	M	ID, BD	AP: RISP, ARP, QTP, CMZ	–	Normal, EM
				Other: VPA		

*ASD, autism spectrum disorder; BD, behavioral disorder; BPD, borderline personality disorder; COS, childhood onset schizophrenia; ID, intellectual disability; ODD, oppositional defiant disorder; PTSD, posttraumatic stress disorder; AP, antipsychotic; AD, antidepressant; AMS, amisulpride; ARP, aripiprazole; BZD, benzodiazepine; CBZ, carbamazepine; CLZ, clozapine; CMZ, cyamemazine; ESC, escitalopram; FLX, fluoxetine; HAL, haloperidol; Li, lithium salt; LMZ, levomepromazine; LOX, loxapine; OLZ, olanzapine; PCZ, propericiazine; PMZ, pimozide; QTP, quetiapine; RISP, risperidone; SRT, sertraline; VPA, valproic acid; ZP, zuclopenthixol; EM, extensive metabolizer; PM, poor metabolizer; UM, ultrarapid metabolizer*.

Five of the nine (55.6%) inpatients with pharmacoresistant mental health disease presented functional CYP2D6 abnormalities. Gene duplication associated with UM has been found in three patients. Two patients were shown to present PM as the consequence of *4/*41 and *3/*4 polymorphisms.

Major adverse events were described in 4/9 patients representing 1/2 of PM and 2/3 of UM (see Table 2).

## Discussion

More than half of our inpatient sample with pharmacoresistant mental health disease presented functional defects in CYP2D6 drug metabolism. This result is somewhat surprising with regard to two aspects. First, we did not expect such a high percentage in our patients. Even if those patients presented with severe mental illness with repeated treatment failure, we attributed treatment refractoriness to diagnosis such as COS, known to be associated with failure of AP treatment (19). In addition, *CYP2D6* anomalies don’t explain resistance to both 2D6 and non-2D6 metabolized drugs.

Second, treatment resistance is generally associated with UM and adverse advents with PM (4, 8). Nevertheless, in our sample both, PM and UM, functional abnormalities are present and no clear relation can be established with the occurrence or absence of major adverse events. Also, whereas one cannot draw conclusions because of the small sample size, the complexity of individual drug metabolism beyond CYP2D6 metabolism needs to be considered for the interpretation of the results. In fact, the accumulation of metabolites, dependent on individual PGx, might explain some side effects. This might be comparable to morphine accumulation leading to intoxication upon codeine treatment in individuals presenting CYP2D6 UM (20, 21). Nevertheless, pharmacokinetics and pharmacodynamics of psychotropic drugs are not yet fully understood, and include additional CYP450 enzymes (e.g., CYP1A2, 3A4/5, 2C9, 2C19) as well as transporter and receptor genes (22). Moreover, CYP expression patterns alter with age and might thus modify the relative contribution of the different enzymes for drug metabolism in children compared to adult patients (23).

In addition to direct metabolism by CYP2D6, some AP (e.g., risperidone) and AD drugs (e.g., fluoxetine) are inhibitors of CYP2D6 or other CYP, and therefore important for treatment interaction which might also be related to pharmacoresistance or side effects (see Table 1).

Moreover, CYP2D6 function outside the hepatic pathway still needs to be elucidated. CYP2D6 has been described to be expressed in the brain, playing a role in acute and chronic drug response as well as in neurotransmitter formation (24). In addition, CYP might be associated with mental health disorders (25, 26). The impact of abnormal function of CYP in pediatric patients with ongoing neurodevelopment might therefore be beyond the only hepatic level.

The knowledge of the individual metabolism of psychotropic drugs is very important for treatment decisions, especially for pharmacoresistant patients. Therapy in line with PGx in those patients should improve treatment outcome and decrease the burden of chronic mental health disease and eventual adverse events in line with predictable inefficient treatment and often polypharmacy. In fact, it has been shown that carriers of abnormal CYP2D6 phenotypes (PM as well as UM) experience an increased duration of hospitalization in psychiatry compared to patients with normal metabolism (27). Nevertheless, so far there are no studies in CAP providing evidence of better treatment outcome when using PGx, and our case descriptions should therefore be interpreted with caution.

In clinical practice, pharmacoresistant UM patients might benefit from increased dosage of CYP2D6-metabolized psychotropics (3). Nevertheless, in the case of adverse events or pharmacoresistance related to PM, treatment options should consider psychotropics not metabolized by CYP2D6 [e.g., olanzapine, clozapine metabolized by CYP1A2; lithium, amisulpride without hepatic metabolism (3), see also http://www.hug-ge.ch/sites/interhug/files/structures/pharmacologie_et_toxicologie_cliniques/a5_cytochromes_hd.pdf]. In general, the clinical benefit of CYP2D6 metabolized molecules should be carefully re-evaluated in patients with abnormal metabolism. Some alternative treatments might not have market authorization in pediatric patients, and should therefore be discussed by a specialized multidisciplinary team. Treatment adjustment should also consider the intensification of non-pharmacological treatments such as psychotherapy.

## Conclusion

Functional anomalies of CYP genotypes might concern a significant number of treatment resistant children and adolescents with severe mental health disorder. Nevertheless, additional studies are needed in order to better understand nonresponding in this population. Considering PGx testing might therefore be important for the clinical workup and treatment decisions in those patients. In fact, the knowledge of individual metabolism should permit better treatment outcome, increased adherence and diminished adverse events (22, 28).

## Perspectives

Our preliminary data underline the need of larger controlled studies of PGx factors in pharmacoresistant children and adolescents in CAP. They should include a broader set of pharmacokinetic and pharmacodynamics parameters, as well as detailed assessment of diagnosis, clinical and treatment history. Moreover, studies are needed to provide evidence of better treatment outcome when using PGx.

A prospective study including children and adolescent with pharmacoresistance to two or more AP and/or AD metabolized by CYP2D6 has been implemented in our department following the preliminary results described in this study. Pharmacoresistance has been defined based on guidelines of the European Medicines Agency (EMA) as lack of satisfactory improvement despite the use of adequate doses of at least two different AP/AD agents, prescribed for adequate duration with adequate confirmation of treatment adherence (EMA/CHMP/40072/2010; EMA/CHMP/185423/2010). PGx explorations will include pharmacokinetic and pharmacodynamic factors, such as *CYP2D6* but also additional CYP450 enzymes as well as transporter and receptor genes. In addition to the results of classic *CYP2D6* genotyping used in our patients, next-generation sequencing will allow rapid DNA sequencing of various genes and thus permit to find additional rare polymorphisms not being discovered with the standard technique.

## Ethics Statement

All patients/guardians gave written informed consent for CYP450 PGx testing and for the use of these data for research purposes and publication. The data collection has been declared to the French national commission on informatics and liberty (CNIL No. 1970115v0).

## Author Contributions

ST, ED, GL, MB, AD, and FA were involved in clinical follow-up. CV has been responsible for pharmacogenetic studies. ST, RD, FA, and CV were responsible for data analysis. ST and CV have been involved in drafting the first version of the MS. All authors have been involved in revising it critically for important intellectual content and approved the final version.

## Conflict of Interest Statement

The authors declare that the research was conducted in the absence of any commercial or financial relationships that could be construed as a potential conflict of interest.

## Footnotes

^1^https://www.pharmvar.org/gene/CYP2D6.

^2^https://www.pharmvar.org/gene/CYP2C19.
